# Hospital GamePlan4Care: Iterative Adaptation of a Caregiver Support Intervention for Hospital Care

**DOI:** 10.1093/geroni/igaf122.037

**Published:** 2025-12-31

**Authors:** Molly Horstman, Tracy Evans, Timothy Zhou, Aanand Naik, Alan Stevens, Mark Kunik

**Affiliations:** Michael E. DeBakey VA Medical Center, Houston, Texas, United States; Baylor College of Medicine, Houston, Texas, United States; Baylor College of Medicine, Houston, Texas, United States; UTHealth Houston, Houston, Texas, United States; Baylor Scott & White Health, Belton, Texas, United States; Baylor College of Medicine, Houston, Texas, United States

## Abstract

There are no evidence-based support interventions tailored to the needs of caregivers of hospitalized adults with dementia. Planned adaptation is an iterative approach that improves the fit between an intervention and a new implementation setting. Our objective is to describe the pre-implementation and post-implementation adaptation of GamePlan4Care for the hospital setting (Hospital GamePlan4Care). GamePlan4Care is an evidence-informed intervention that provides tailored education, support, and skills training to caregivers of adults with dementia in the community. Pre-implementation adaptations were guided by a literature review, semi-structured interviews with caregivers and health professionals, and a stakeholder panel that included caregivers. Post-implementation adaptations were guided by semi-structured interviews with caregiver participants who completed a single-arm pilot study of Hospital GamePlan4Care. Pre-implementation adaptations focused on intervention content: adding new content and modifying existing content to improve accessibility. Caregivers who completed the pilot study found the content helpful but faced challenges finding time to meet with the interventionist and complete the online training. Based on caregiver feedback, adaptations were made to improve the fit between the intervention and the lived experience of caregivers following discharge. Post-implementation adaptations included reducing the length of the intervention and creating greater flexibility in the timing and mode of therapeutic sessions. Caregiver feedback informed adaptions to the content and intervention delivery. Piloting the intervention enabled caregivers to provide concrete recommendations to improve the fit between the intervention and the caregiver lived experience of the hospitalization and return to home-based care.

